# Supplementary material for: COVID-19 Attitudes and Vaccine Hesitancy among an Agricultural Community in Southwest Guatemala: A Cross-Sectional Survey
Source: Vaccines (Basel). 2023 Jun 2;11(6):1059. doi: 10.3390/vaccines11061059 (PMC10302313; doi:10.3390/vaccines11061059)
Supplement: Supplementary file 1 [file vaccines-11-01059-s001.zip › vaccines-2366356-supplementary.pdf]

Actitudes sobre el COVID-19 e indecisión respecto a la vacuna en una comunidad agrícola en el suroeste de Guatemala: una encuesta transversal

Neudy Rojop [1], Diva M. Calvimontes [1,2], Edgar Barrios [1], Molly M. Lamb [2,3], Alejandra Paniagua-Avila [4], Jose Monzon [5], Lindsey M. Duca [5], Chelsea Iwamoto [5], Anna N. Chard [5], Melissa Gomez [1], Kareen Arias [1], Yannik Roell [2], Guillermo Antonio Bolanos [1], Emily Zielinski-Gutierrez [5], Eduardo Azziz-Baumgartner [5], Maria Renee Lopez [6], Celia Cordon-Rosales [6], Edwin J. Asturias [1,2,3,7] and Daniel Olson [1,2,3,7,*]

[1] Center for Human Development, Fundación para la Salud Integral de los Guatemaltecos, Retalhuleu 11010, Guatemala; rojophernandez@gmail.com (N.R.); diva.barrientos@cuanschutz.edu (D.M.C.); ebarrios1908@gmail.com (E.B.); melissa.gomez.fsigcu@gmail.com (M.G.); kareen.arias@gmail.com (K.A.); antonio.bolanos@cuanschutz.edu (G.A.B.); edwin.asturias@cuanschutz.edu (E.J.A.)
[2] Center for Global Health, Colorado School of Public Health, Aurora, CO 80045, USA; molly.lamb@cuanschutz.edu (M.M.L.); yannik.roell@cuanschutz.edu (Y.R.)
[3] Department of Epidemiology, Colorado School of Public Health, Aurora, CO 80045, USA
[4] Department of Epidemiology, Mailman School of Public Health, Columbia University, New York, NY 11032, USA; mp3856@cumc.columbia.edu
[5] Centers for Disease Control and Prevention (CDC), Atlanta, GA 30333, USA; rgi4@cdc.gov (J.M.); pgz5@cdc.gov (L.M.D.); oeu5@cdc.gov (C.I.); mmn9@cdc.gov (A.N.C.); ebz0@cdc.gov (E.Z.-G.); eha9@cdc.gov (E.A.-B.)
[6] Centro de Estudios en Salud, Universidad del Valle de Guatemala, Guatemala City 01015, Guatemala; mlopez@ces.uvg.edu.gt (M.R.L.); ccordon@ces.uvg.edu.gt (C.C.-R.)
[7] Department of Pediatrics, University of Colorado School of Medicine, Aurora, CO 80045, USA
* Correspondence: daniel.olson@cuanschutz.edu; Tel.: +720–777−6981

Resumen

A pesar de que a partir de julio del 2021 ofrece vacunas contra el COVID-19 gratuitas, Guatemala tiene una de las tasas de vacunación más bajas en Latinoamérica. Desde el 28 de septiembre del 2021 hasta el 11 de abril del 2022, hicimos una encuesta transversal a miembros de la comunidad, en la que adaptamos un cuestionario de los CDC, para evaluar el acceso a la vacuna contra el COVID-19 y la indecisión respecto a la vacuna. De los 233 participantes de ≥12 años, 127 (55 %) habían recibido ≥1 dosis de la vacuna contra el COVID-19 y 4 (2 %) notificaron haber tenido previamente la enfermedad. Era más probable que las personas de ≥12 años no vacunadas (n=106) fueran mujeres (73 % vs. 41 %, p<0.001) y amas de casa (69 % vs. 24 %, p<0.01), en comparación con los participantes vacunados (n=127). Entre las personas de ≥18

años, la principal motivación para vacunarse reportada entre los participantes vacunados fue proteger la salud de la familia/amigos (101/117, 86 %); 40 personas no vacunadas (55 %) reportaron tener poca confianza/nada de confianza en las instituciones de salud pública que recomiendan la vacunación contra el COVID-19. Los programas de vacunación basados en la comunidad o en el hogar, incluida la vacunación de familias a través del lugar de trabajo, podrían llegar mejor a las mujeres que son amas de casa, y reducir las inequidades y la indecisión. (S1: manuscrito traducido al español).

Introducción

A pesar de la carga significativa que tiene el COVID-19, a nivel económico y como enfermedad, y de que la vacunación es gratuita, los países de bajos y medianos ingresos han tenido dificultades para lograr altas tasas de vacunación. El acceso limitado a las vacunas y, cada vez más, la indecisión respecto a la vacuna provocan reiteradas olas de transmisión comunitaria y desperdician recursos valiosos.[1, 2]

El acceso a las vacunas y la renuencia a las vacunas son dos fenómenos distintos, pero superpuestos, que se han identificado como barreras para la vacunación mundial en general y la vacunación contra COVID-19 en particular. [3,4] Muchas poblaciones tanto en los países de ingresos altos (HIC) como en los LMIC enfrenta barreras constantes para el acceso a las vacunas, con una menor asignación de vacunas y una infraestructura logística y de salud pública más deficiente; estas barreras se vieron exacerbadas por la pandemia de COVID-19. [3,5,6]

Además, tanto los países de ingresos altos con mayor acceso a vacunas efectivas, como los LMIC cada vez más, se han visto afectados por la indecisión ante las vacunas. [4,7] El grupo de trabajo sobre la reticencia a la vacunación del Grupo Estratégico Consultivo de Expertos (SAGE) de la Organización Mundial de la Salud (OMS) define la reticencia a la vacunación como "un retraso en la aceptación o el rechazo de la vacunación a pesar de la disponibilidad de servicios de vacunación" [8]. Es importante señalar que la indecisión antes las vacunas es "compleja y específica del contexto", "varía en función del tiempo, el lugar y las vacunas" e "influye la complacencia, la conveniencia y la confianza". Por lo tanto, abordar el acceso a las vacunas y la indecisión requieres una comprensión dinámica de contexto local.

Guatemala es uno de estos países, con un historial de acceso inconsistente a las vacunas y una alta aceptación de las mismas, aunque la indecisión ante las vacunas en ciertas poblaciones está

aumentando, específicamente durante la pandemia de COVID-19. [9-13] El sistema sanitario de Guatemala se compone de dos sectores, público y privado. El primero comprende el Ministerio de Salud Pública y Asistencia Social (MSPAS), que cubre al 70% de la población, incluido su programa de inmunización, el Instituto Guatemalteco de Seguridad Social (IGSS), que da cobertura al 18% de la población vinculada al empleo formal, y el Servicio de Sanidad Militar, que presta servicios sanitarios a los miembros de las fuerzas armadas y la policía (menos del 0,5%). El sector privado incluye organizaciones de la sociedad civil y/o religiosas que operan sin ánimo de lucro, además de varios proveedores con ánimo de lucro. El MSPAS se financia con recursos procedentes de los impuestos estatales, ayudas, préstamos y donaciones internacionales.

Guatemala comenzó su programa de vacunación contra el COVID-19 el 25 de febrero del 2021, dirigido a los trabajadores de la salud y a personas con enfermedades concomitantes; los adolescentes de 12 a 17 años fueron incluidos a partir del 22 de septiembre del 2021. A pesar de la cada vez mayor disponibilidad de vacunas contra el COVID-19 gratuitas, la cobertura en Guatemala sigue siendo una de las más bajas en Latinoamérica, especialmente en las comunidades rurales e indígenas. Hasta el 1.º de julio del 2022, solo el 46 % de la población elegible en Guatemala había recibido una dosis de la vacuna y el 35 % había recibido dos dosis.[3, 4] Para abril del 2022, 1.47 millones de dosis de la vacuna contra el COVID-19 donadas por los Estados Unidos[5] y casi 5 millones de dosis compradas de la vacuna Sputnik V[6] habían vencido, supuestamente debido a la baja accesibilidad y demanda en todo el país.

Aunque existen datos limitados sobre la prevalencia cambiante de la indecisión y el acceso a las vacunas en toda Guatemala, especialmente en el contexto de la pandemia de COVID-19, algunas poblaciones no se han caracterizado bien. Una población crítica es la de los

trabajadores agrícolas esenciales, que representan el 35% de la mano de obra total de Guatemala y el 11,3% de su producto interior bruto [18]. Al igual que en muchos PIBM, los trabajadores agrícolas guatemaltecos no sólo desempeñan un papel fundamental en el suministro local de alimentos, sino también en la seguridad alimentaria mundial [19]. [A menudo son los principales generadores de ingresos para sus hogares y, como trabajadores esenciales, siguieron trabajando durante la pandemia a pesar de las altas tasas de infección por SARS-CoV-2 [20,21]. Por lo tanto, comprender la dinámica y los factores que impulsan la no vacunación y la indecisión ante la vacuna en esta población activa es importante para reducir los efectos negativos del COVID-19 y otros patógenos emergentes mediante futuros programas de vacunación.

Por lo tanto, los objetivos de este estudio eran entender la frecuencia y los factores que impulsan la cobertura de la vacunación contra el COVID-19 y la indecisión respecto a la vacuna dentro de una comunidad agrícola en áreas rurales de Guatemala, con el fin de diseñar una intervención para mejorar la cobertura de la vacunación contra el COVID-19 (S1: manuscrito traducido al español).

Métodos

Hicimos una encuesta transversal para evaluar los conocimientos, las actitudes y las prácticas respecto al COVID-19 y las vacunas disponibles de manera gratuita. Esta encuesta se incluyó en la visita de inscripción de un estudio de cohorte prospectivo para caracterizar la transmisión del SARS-CoV-2 asintomática y presintomática en los hogares y los lugares de trabajo de trabajadores agrícolas bananeros. El estudio se hizo entre el 28 de septiembre del 2021

y el 11 de abril del 2022, en dos comunidades rurales (Los Encuentros en Quetzaltenango y Chiquirines en San Marcos) en las tierras bajas del suroeste de Guatemala, aproximadamente a 50 km de la frontera con Chiapas, México. Las comunidades son monolingües de habla hispana. La población padece altas tasas de inseguridad alimentaria y desnutrición infantil durante todo el año, enfermedades diarreicas, depresión materna y morbilidad y mortalidad materna e infantil [22-24]. [22-24] Encuestas anteriores (2015, 2017-18) realizadas entre trabajadores agrícolas en las mismas comunidades encontraron una mano de obra predominantemente joven, masculina y económicamente vulnerable, en la que los trabajadores agrícolas tienden a ser los principales generadores de ingresos para sus hogares; los trabajadores informan de altas tasas de inseguridad alimentaria, pobreza y enfermedades transmisibles, así como de un bajo acceso a la atención sanitaria [25]. La emigración a EE.UU. de esta comunidad es frecuente, incluido el 3% en 2022 de un estudio de cohortes comunitario independiente (sin publicar).

Los criterios de inclusión para el estudio de origen y la encuesta fueron los siguientes: ≥1 miembros del hogar empleados en el sector agrícola, deben vivir en las comunidades seleccionadas, ≥75 % de las personas que viven en la casa deben dar su consentimiento para participar, y al momento de la encuesta deben ser elegibles para recibir las vacunas contra el COVID-19 (edad de ≥12 años; solo es un criterio de inclusión para la encuesta). El cuestionario (S2: versiones en inglés y español de la encuesta) incluyó la encuesta adaptada en español "Confianza en la vacuna contra el COVID-19", hecha por los Centros para el Control y la Prevención de Enfermedades (CDC),[7] La encuesta incluía las siguientes áreas temáticas y preguntas datos demográficos (edad, sexo, origen étnico); entorno laboral (ocupación, lugar de

trabajo); enfermedades preexistentes (asma, enfermedad pulmonar, enfermedad renal, enfermedad cardiaca, diabetes, trastorno sanguíneo, enfermedad neurológica y enfermedad hepática); conocimientos, actitudes y prácticas en relación con COVID-19 (CAP; exposición previa, nivel de preocupación, pruebas de confirmación, uso de equipo de protección individual [EPI]); historial de vacunación contra la COVID-19 (fecha(s), tipo(s) de vacuna(s), número de dosis); historial de rechazo a la vacunación y motivo(s) para ello; historial de no acceso a las vacunas y motivo(s) para ello. La encuesta también recogía preguntas específicas sobre la indecisión y la toma de decisiones en relación con la vacuna COVID-19, entre las que se incluían las siguientes: percepción de la seguridad de la vacunación, preferencias en cuanto a la vacunación (lugar), motivaciones para la vacunación, toma de decisiones entre iguales en relación con la vacunación COVID-19, uso de EPP si se vacuna, confianza en las instituciones de salud pública, cuantificación de la información disponible sobre la vacunación COVID-19 y exposición a información errónea sobre la vacuna COVID-19. Así como preguntas adicionales sobre características demográficas, estado de vacunación, y conocimientos, actitudes y prácticas sobre el COVID-19, las vacunas contra el COVID-19 y las vacunas en general. La encuesta fue hecha verbalmente a los adultos y niños (acompañados por adultos) por miembros del personal de enfermería del estudio capacitados, en los hogares o lugares de trabajo de los participantes, y las respuestas fueron grabadas usando la aplicación REDCap (University de Colorado Anschutz Medical Campus, Aurora CO, EE.UU.) en un *smartphone*.

Al momento de la encuesta, solo se ofrecía la vacunación contra el COVID-19 en puestos del Ministerio de Salud (por lo general dos días a la semana) y a través de programas de

vacunación en el lugar de trabajo por el sistema del Instituto Guatemalteco de Seguridad Social (IGSS), como el de la empresa agrícola que empleaba a por lo menos un miembro de cada hogar. Las vacunas disponibles en Guatemala, al momento de la encuesta, incluían la mRNA-1273 (mRNA, Moderna, Cambridge, EE. UU.), BNT162b2 (mRNA, Pfizer-BioNTech, Nueva York, EE. UU.), ChAdOx1-S (vector viral, AstraZeneca, Cambridge, Inglaterra) y Gam-COVID-Vac (vector de virus, Sputnik V, Rusia). Todas estas vacunas requieren dos dosis en la serie primaria. En Guatemala, las personas de ≥12 años se volvieron elegibles para recibir la vacuna contra el COVID-19 en septiembre del 2021. Los datos de vacunación se obtuvieron directamente del registro nacional de vacunación y se verificaron a través del autorreporte de los participantes.

Se usó el *software* SPSS® (versión 25, Chicago, Il, EE. UU.) para hacer el análisis de los datos. Se consideró vacunados a los participantes con ≥1 dosis de la vacuna contra el COVID-19. Se usaron estadísticas descriptivas para caracterizar las respuestas de la encuesta. Se usó la prueba de la *U* de Mann-Whitney para las comparaciones de medianas, y la prueba de la $\chi 2$ de Pearson y la prueba exacta de Fisher para las proporciones; un valor p <0.05 se consideró estadísticamente significativo. El estudio fue aprobado por la Universidad de Colorado (COMIRB, protocolo #21-2551), la Universidad del Valle de Guatemala (UVG) y comités de ética de los CDC; fue financiado por los CDC (CDCGH002243).

Resultados

Desde el 28 de septiembre del 2021 hasta el 11 de abril del 2022, inscribimos a 340 personas (el 86 % de las 394 personas elegibles) de 74 hogares (véase el mapa, figura 1); 233 personas (69 %) tenían ≥12 años y 190 personas (56 %) tenían ≥18 años (Tabla 1). Los hogares y

las personas de las comunidades de Chiquirines y Los Encuentros eran similares en cuanto a las características demográficas y los riesgos de exposición, aunque la media de ingresos mensuales de los hogares era ligeramente superior en Chiquirines ($USD 250 frente a $USD 197,37, p =

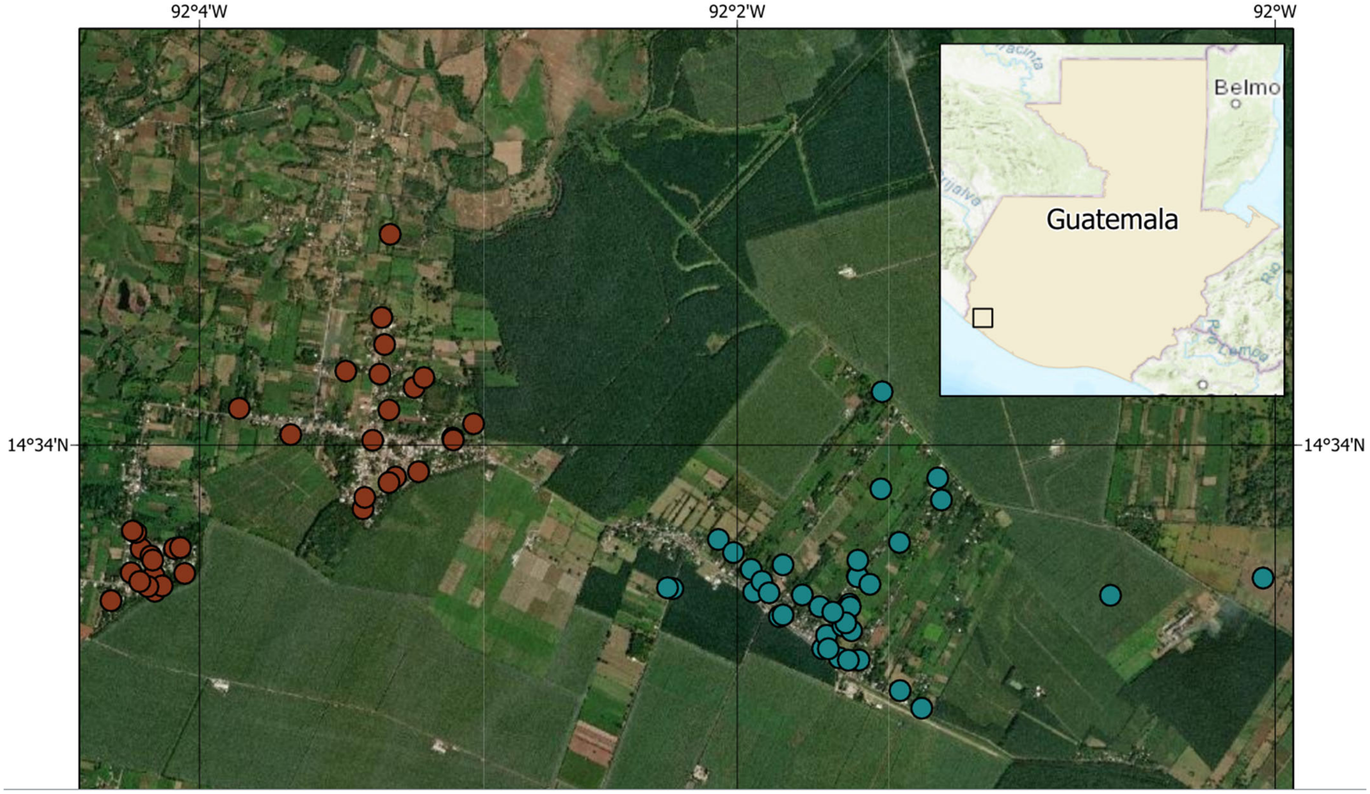

0,04). En general, la mediana de ingresos familiares mensuales era de $210.73 =quartile deviation (QD) = $92.11). De las 340 personas inscritas, 177 (52 %) eran mujeres, 323 (95 %) eran de etnicidad ladina/mestiza (mezcla de españoles e indígenas), y de aquellas personas de >15 años, 107 (53 %) trabajaban fuera del hogar. La mediana de edad fue de 21 años (QD=12.25, rango=0-73 años). Ningún niño reportó asistir a la escuela, ya que todas las escuelas en la comunidad estaban cerradas debido a la pandemia de COVID-19.

Figura 1. Mapa que muestra las áreas de captación del estudio y los hogares de los municipios Chiquirines y los Encuentros que se incluyeron en la encuesta de indecisión a la vacuna, 28 de septiembre de 2021 a 11 de abril de 2022.

Tabla 1. Características sociodemográficas de los hogares inscritos en dos comunidades rurales en la Región del Trifinio del suroeste de Guatemala, 2021-2022.

Variable	Los Encuentros (n=40)	Chiquirines (n=34)	Valor *p***
**Hogar**			
Personas que viven en el hogar, mediana (QD)	5 (1)	5 (1)	0.76
Niños que viven en el hogar, mediana (QD)	2(1)	3 (1)	0.22
Camas en el hogar, mediana (QD)	3 (1)	3 (1.5)	0.90
Ingresos familiares mensuales, $USD, mediana (QD) *	197.37 (65.8)	260 (72.37)	0.04
**A nivel individual**	**(n=181)**	**(n=159)**	
Edad, mediana (DE)	21 (6.5)	20 (12.5)	0.93
Mujeres, n (%)	95 (52)	82 (52)	0.86
Etnicidad			
Ladina/mestiza**, n (%)	166 (92)	157 (99)	< 0.01
Indígena	3 (2)	1 (0.5)	
No sabe	12 (6)	1 (0.5)	
Reporta comorbilidad, n (%)	33 (18)	20 (13)	0.15
Trabaja fuera del hogar, tiene ≥15 años, n (%)	53/112 (47)	54/90 (60)	0.29
Vacuna contra el COVID-19, tiene ≥12 años, n (%)	67/123 (54)	60/110 (55)	0.99

Abreviaturas: QD = desviación de cuartil, * Tipo de cambio: $1 USD (1 US dollar) = Q7.6 (7.6 Guatemala quetzales). **Descendiente de mezcla de españoles e indígenas. *****Valor *p* =** se usó la prueba de la *U* de Mann-Whitney para las comparaciones de medianas, y la prueba de la $\chi 2$ de Pearson y la prueba exacta de Fisher para las proporciones; un valor *p* <0.05 se consideró estadísticamente significativo en todas las comparaciones.

Al momento de la encuesta, 4 encuestados (2 %) reportaron haber tenido COVID-19 previamente; 127 (55 %) reportaron haber recibido ≥1 dosis de una vacuna contra el COVID-19, lo cual incluyó mRNA-1273 (n=106, 83 %), ChAdOx1-S (n=15, 12 %), BNT162b2 (n=4, 3 %), Gam-COVID-Vac (n=1, 1 %) y Cansino (n=1, 1 %; administrada en la zona cercana de México). De

ellos, 89 (70%) declararon haber recibido dos dosis de la vacuna COVID-19, y la mayoría (n=88, 97%) recibieron la vacuna ARNm-1273. Sólo dos sujetos (2%) declararon haber recibido una tercera dosis de vacuna, que incluía vacunas de ARNm. Solo 11 participantes (5 %) reportaron haber rechazado alguna vez una vacuna en el pasado; las razones comunes de ese rechazo en el pasado fueron pensar que la vacuna no era necesaria (n=7, 64 %), preocupación por los efectos secundarios (n=2, 18 %) y que otra persona le dijera al encuestado que la vacuna no era segura (n=2, 18 %), (Tabla 2).

Cuarenta (17,2%) de los encuestados declararon no haber podido vacunarse anteriormente a pesar de haberlo intentado. Las razones más comunes fueron que la vacuna no estaba disponible (n = 14, 32%), no saber dónde vacunarse (n = 7, 16%), no poder salir del trabajo en horario de clínica (n = 5, 12%) y no saber dónde obtener información buena y fiable sobre la vacuna (n = 5, 12%).

Tabla 2. Datos sobre la vacunación contra el COVID-19 e indecisión respecto a la vacuna obtenidos de los encuestados elegibles para recibir la vacuna.

**Vacunación contra el COVID-19 en participantes de ≥12 años.**	**n (233)**
Recibió la vacuna contra el COVID-19* n (%)	127 (55)
Recibió 1 dosis de la vacuna contra el COVID-19, n (%)	36 (28)
Recibió 2 dosis de la vacuna contra el COVID-19, n (%)	89 (70)
Recibió 3 dosis (con dosis de refuerzo) de la vacuna contra el COVID-19, n (%)	2 (2)
Primera dosis de la vacuna contra el COVID-19 (n=127)	
Moderna, n (%)	106 (83)
AstraZeneca, n (%)	15 (12)
Pfizer-BioNTech, n (%)	4 (3)
Sputnik V, n (%)	1 (1)
Otra (Cansino), n (%)	1 (1)
Segunda dosis de la vacuna contra el COVID-19 (n=91)	

Moderna, n (%)	88 (97)
AstraZeneca, n (%)	2 (2)
Sputnik V, n (%)	1 (1)
Tercera dosis (dosis de refuerzo) de la vacuna contra el COVID-19 (n= 2)	
Moderna, n (%)	1 (50)
Pfizer-BioNTech, n (%)	1 (50)
**Indecisión respecto a la vacuna**	
Antecedentes de COVID-19 (autorreportados), n (%)	4 (2)
Se negó a recibir una vacuna de rutina previamente, n (%)	11 (5)
Razones para negarse a recibir la vacuna (n=11)*	**n (%)**
No pensé que fuera necesaria, n (%)	7 (63)
Me preocupaban los efectos secundarios, n (%)	2 (18)
Otra persona me dijo que la vacuna no era segura, n (%)	2 (18)
Otra, n (%)	3 (27)
Previamente (antes de la pandemia), quiso recibir una vacuna de rutina (cualquier tipo), pero no pudo recibirla, n (%)	40 (17)
Razones más comunes para no haber podido, previamente, recibir una vacuna de rutina a pesar de tener la intención (n=40)	**n (%)**
La vacuna no estaba disponible en mi centro de salud ni en mi comunidad	14 (32)
No sabía dónde ir para vacunarme	7 (16)
No sabía dónde obtener información buena y confiable sobre la	5 (12)
vacuna	1 (2)
No podía pagar por la vacuna	5 (12)
No podía dejar mi trabajo para recibir la vacuna durante el horario del centro médico	11 (26)
Otra razón	

* Se consideró vacunada a una persona con $\geq$1 dosis de la vacuna contra el COVID-19.
** Puede elegir más de una respuesta.

Al comparar individuos vacunados y no vacunados > 12 años (elegibles) en el momento de la encuesta era más probable que las personas vacunadas fueran hombres (59 % vs. 27 %, p <0.01), de mayor edad (mediana de edad = 33 vs. 25 años, p <0.01), que trabajaran fuera de la casa (69 % vs. 24 %, p <0.01) o que trabajaran en una granja (70 % vs. 43 %, p=0.008) (Tabla 3, figura 2). De las personas vacunadas, 4 (3 %) reportaron haber rechazado otro tipo de vacunación previamente (antes de la pandemia de COVID-19) vs. 7 (7 %) de las personas no

vacunadas (*p*=0.23). No hubo diferencias entre las personas vacunadas y las no vacunadas en cuanto a los motivos por los que no pudieron acceder a las vacunas en el pasado.

Entre las personas de ≥18 años (Tabla 3), al momento de la encuesta, era más probable que las personas vacunadas estuvieran moderadamente o muy preocupadas por el COVID-19 (n=36, 31 %) en comparación con las personas no vacunadas (n=13, 18 %; *p*=0.04). Las motivaciones más frecuentes reportadas para la vacunación contra el COVID-19 se muestran en la Tabla 3 e incluyen proteger la salud del participante, lo cual difería entre las personas vacunadas y las no vacunadas (24 % vs. 11 %, respectivamente; *p*<0.01); no había diferencia entre la motivación relacionada con proteger a su familia/amigos (68 % vs. 73 %, *p*=0.53), proteger a su comunidad (3 % vs. 3 %, *p*=0.94), volver al trabajo o a la escuela (2,5% frente a 3, p = 0,94), o ser animado por otros a vacunarse (2% frente a 0%, p = no calculable). En comparación con las personas vacunadas, era más probable que las no vacunadas reportaran tener poca confianza/nada de confianza en las instituciones de salud pública (38 % vs. 55 %, *p*=0.02). Entre los 73 participantes no vacunados >18 años (38 %), el 25 % reportó que se pondría la vacuna lo antes posible, el 40 % reportó que se pondría la vacuna pero esperaría, el 11 % respondió que no se pondría la vacuna y el 25 % reportó no estar seguro.

Tabla 3. Comparación de participantes de ≥ 12 años vacunados y no vacunados contra el COVID-19.

Variable	Vacunado n=127 (%)	No vacunado n=106 (%)	Valor *p***
Edad, mediana (QD)	33 (7)	25 (10)	<0.01
12 – 17	10 (8)	33 (31)	<0.01
18 – 30	44 (35)	37 (35)	0.96
31 – 40	42 (33)	19 (18)	<0.01
≥41	31 (24)	17 (16)	0.11
Mujer, n (%)	52 (41)	77 (73)	<0.01
Ladino/mestizo, n (%)	122 (96)	98 (93)	0.45
Trabaja fuera del hogar, n (%)	87 (69)	25 (24)	<0.01

Comorbilidad presente, n (%)*	30 (24)	17 (16)	0.15
¿Se negó a recibir una vacuna previamente n (%)	4 (3)	7 (7)	0.23
Razones para negarse a recibir la vacuna (puede seleccionar >1)	n=4	n=7	
No pensé que fuera necesaria	2 (50)	5 (71)	0.57
No sabía dónde obtener información confiable	1 (25)	0 (0)	n/a
Me preocupaban los efectos secundarios	0 (0)	2 (29)	n/a
Otra persona me dijo que la vacuna no era segura	0 (0)	2 (29)	n/a
Miedo a las agujas	0 (0)	1 (14)	n/a
No pude salir del trabajo/casa para ir a vacunarme	1 (25)	0 (0)	n/a
Previamente (antes de la pandemia), quiso recibir una vacuna (cualquier tipo), pero no pudo recibirla	19 (15)	21 (20)	0.32
Razones por las que no pudo vacunarse (antes de la pandemia, puede seleccionar >1)	n=19	n=21	
La vacuna no estaba disponible en mi centro de salud ni en mi comunidad	5 (26)	9 (43)	0.27
No sabía dónde ir para vacunarme	3 (16)	4 (19)	1.00
No sabía dónde obtener información buena y confiable sobre la vacuna	2 (11)	3 (14)	1.00
No podía pagar por la vacuna	1 (5)	0 (0)	n/a
No podía dejar mi trabajo para recibir la vacuna durante el horario del centro médico	4 (21)	1 (5)	0.17
Otra barrera para recibir la vacuna	4 (21)	7 (33)	0.48
**Participantes de ≥ 18 años**	**n= 117 (%)**	**n=73 (%)**	
¿Qué tan preocupado está o estaba de contraer el COVID-19?			
Para nada preocupado	35 (30)	33 (45)	0.32
Un poco preocupado	46 (39)	27 (37)	0.74
Moderadamente preocupado	14 (12)	7 (10)	0.61
Muy preocupado	22 (19)	6 (8)	0.04
Principal motivación para vacunarse contra el COVID-19			
Proteger mi salud	28 (24)	8 (11)	0.02
Proteger la salud de mi familia y mis amigos	80 (68)	53 (73)	0.53
Proteger la salud de mi comunidad	3 (2.5)	2 (3)	0.94
Regresar al trabajo o a la escuela	3 (2.5)	0 (0)	n/a
Otras personas me alentaron a vacunarme	2 (2)	0 (0)	n/a
Otra	1 (1)	0 (0)	n/a
No estoy seguro	0 (0)	10 (13)	n/a
Poca confianza o nada de confianza en las instituciones de salud pública que recomiendan la vacunación	44 (38)	40 (55)	0.02
Vio o escuchó información sobre las vacunas contra el COVID-19 (noticias, redes sociales, amigos y familiares)	92 (79)	50 (69)	0.25
No recibió suficiente información sobre las vacunas contra el COVID-19	14 (12)	21 (29)	0.01
Sabe dónde conseguir información precisa y oportuna sobre las vacunas contra el COVID-19	80 (68)	42 (57)	0.25

** Comorbilidad se refiere a los participantes que reportaron tener al menos una de las siguientes afecciones: asma, enfermedad pulmonar, enfermedad renal, enfermedad cardiaca, diabetes, anemia, enfermedad neurológica y enfermedad hepática.*

*** Valor p:* se usó la prueba de la *U* de Mann-Whitney para las comparaciones de medianas, y la prueba de la $\chi 2$ de Pearson y la prueba exacta de Fisher para las proporciones; un valor p <0.05 se consideró estadísticamente significativo en todas las comparaciones.

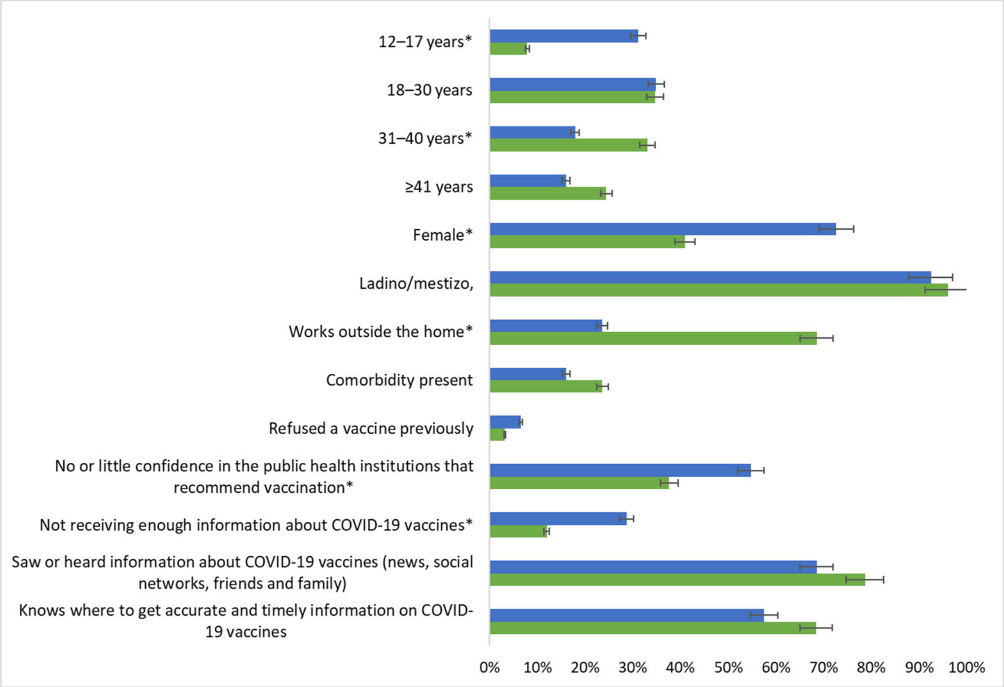

Figura 2. Comparación de miembros de la comunidad vacunados y no vacunados contra COVID-19 en la Región Trifinio del suroeste de Guatemala. *Considerado estadísticamente significativo (valor p < 0,05).

Discusión

Esta encuesta transversal muestra que, aunque el rechazo a las vacunas antes de la pandemia de COVID-19 era raro (5 %), en estas dos comunidades agrícolas de Guatemala, casi la mitad (45 %) de los participantes elegibles para la vacuna contra el COVID-19 permanecieron no vacunados contra esta enfermedad (con ninguna dosis) un año después de que la vacuna contra el COVID-19 estuvo disponible. Esto plantea la pregunta de cómo la distribución e información de la vacuna contra el COVID-19 pudo haber sido distinta en esta comunidad en comparación con programas de vacunación previos, lo que posiblemente contribuyó a una menor aceptación, utilización y confianza.

La mayoría de los encuestados que todavía no se habían vacunado eran mujeres amas de casa. Muchos de los hombres adultos en estas comunidades estaban empleados en grandes empresas agrícolas que ofrecían la vacunación contra el COVID-19 recomendada a través del lugar de trabajo, lo que parece indicar que un mayor acceso a la vacuna (a través del lugar de trabajo) podría mejorar la cobertura de la vacunación. En otros PBI y PIM también se observaron mayores tasas de vacunación e intención de vacunación entre los hombres [27,28], aunque se necesita más información sobre las razones de las mayores tasas de vacunación en estos otros entornos [25]. Otros estudios de América Latina también encontraron barreras estructurales como una razón común para la no vacunación contra COVID-19, destacando la interacción entre el acceso a la vacuna y la indecisión. [29] Ampliar estos programas en el lugar de trabajo a los miembros de la familia de los empleados e implementar intervenciones basadas en el hogar o la comunidad (lugares donde se practica la religión o donde se reúne la comunidad) podría aumentar el acceso para aquellas personas que permanecen sin vacunarse.

De hecho, en Gua-temala y en todo el mundo se ha propuesto involucrar a los líderes religiosos para reducir las dudas sobre las vacunas [10,15,30].

También hallamos evidencia de indecisión respecto a la vacuna. Las personas no vacunadas reportaron tener menor confianza en las instituciones de salud pública, no recibir suficiente información sobre las vacunas y no poder encontrar información precisa y oportuna sobre las vacunas contra el COVID-19.

Aunque los datos de los PBI y los PIM son limitados, se observaron resultados similares en un estudio cualitativo de trabajadores agrícolas latinos inmigrantes en California, EE.UU., en el que se descubrió que la desinformación y la falta de confianza en las instituciones son los temas principales que afectan a las actitudes hacia la vacunación [31].

Estos datos indican la necesidad de contar con mensajes de salud pública mejores y más eficaces en esta comunidad y en áreas rurales similares. De hecho, esta necesidad se ve agravada por la actual "infodemia" de desinformación de otras fuentes, lo cual se ha asociado a una mayor indecisión respecto a la vacuna.[2, 9, 10, 11] Es posible que los retrasos en lograr el acceso a la vacuna en estas comunidades rurales haya servido para socavar la aceptación de la vacuna y haya "abierto la puerta" a una mayor desinformación, lo cual contribuyó a una mayor indecisión y menor aceptación cuando la vacuna estuvo más ampliamente disponible. Futuros estudios, que incluyen una repetición de la encuesta sobre las dudas sobre las vacunas en la misma cohorte de hogares, ayudarán a clarificar este asunto y también abordar el cambio de creencias y comportamientos en torno a la vacunación contra COVID-19.

Este estudio es limitado ya que se llevó a cabo durante la visita de inscripción de un estudio de transmisión del SARS-CoV-2 en los hogares, y estuvo restringido a los hogares de los trabajadores empleados en grandes empresas agrícolas, lo cual limitó su generalizabilidad. Sin embargo, los trabajadores agrícolas conforman el 35 % de toda la fuerza laboral en Guatemala y por lo tanto representan una importante subpoblación en la cual estudiar el acceso a las vacunas y la indecisión respecto a ellas. Este grupo también es esencial para la seguridad alimentaria local y mundial. Las entrevistas de investigación cualitativa permitirían una evaluación más exhaustiva de los complejos factores que impulsan las dudas sobre las vacunas y las barreras de acceso en estas comunidades y otras similares. La encuesta también se llevó a cabo en un momento particular y podría no reflejar las cambiantes actitudes hacia las vacunas, aunque tenemos como objetivo abordar esta limitación con encuestas de seguimiento, como se ha indicado anteriormente

Estos hallazgos proveen una oportunidad para implementar mejores mensajes de salud pública basados en la evidencia y estrategias de acceso en la comunidad. Con base en la necesidad de comunicarse a nivel de hogar y para aumentar la participación de la comunidad y el intercambio de información, estamos diseñando una estrategia de mensajes de salud pública que involucre a un grupo de líderes comunitarios respetados (miembros del Consejo Comunitario de Desarrollo [COCODE], personal de enfermería de puestos de salud, parteras, maestros y líderes religiosos, entre otros) que serán capacitados para proporcionar información sobre la vacuna contra el COVID-19 en la comunidad. Simultáneamente, los puestos de salud en la comunidad están implementando un programa de vacunación casa por casa, por parte del

personal de enfermería de salud pública, que podría llegar a la población que no ha sido vacunada a través de sus lugares de trabajo. Después de este proceso, se volverá a hacer esta encuesta para identificar cambios en la indecisión respecto a la vacuna, y su aceptación y utilización.

Financiamiento: este estudio fue financiado por el Acuerdo de Cooperación de los CDC CDCGH002243. El Dr. Olson recibe financiamiento del NIH/NIAID 1K23AI143967 y de la subvención número UL1 TR001082 del NCATS de los NIH para el CTSI de Colorado. Los hallazgos y las conclusiones que aparecen en este informe pertenecen a los autores y no representan necesariamente los puntos de vista oficiales, ni un respaldo, de los Centros para el Control y la Prevención de Enfermedades, del Departamento de Salud y Servicios Humanos ni del Gobierno de los Estados Unidos.

Reconocimientos. Les agradecemos a las siguientes personas o entidades por sus contribuciones a esta investigación: Equipo de Investigación de Trifinio de la Universidad de Colorado, la administración del Centro de Salud Global de la Universidad de Colorado, AgroAmérica. Les agradecemos a las personas de la comunidad del área Trifinio del Suroeste que participaron en esta investigación, a la Oficina Regional de los CDC en Centroamérica y al Grupo de Trabajo Internacional sobre el COVID-19 de los CDC, incluidas Nga Vuong, María Beatriz López Castellanos, María del Rosario Polo.

Contribuciones de los autores: Conceptualización, NR, DMC, EB, MML, EZG, EAB, EJA y DO; Metodología, NR, DMC, EB, MML, JM, LMD, CI, AC, MG, EZG, EAB, EJA y DO; Software, NR; Validación, NR, EB; Análisis formal, NR, MML; Investigación, NR, DMC, EB, MML, APA, JM, LMD, CI, AC, MG, EZG, EAB, EJA y DO; Recursos, NR, MG; Redacción - Preparación del borrador original, NR, DO; Redacción - Revisión y edición, todos los autores; Visualización, NR,

EJA y DO. Redacción - Preparación del borrador original, NR, DO; Redacción - Revisión y edición, todos los autores; Visualización, NR, MML, DO; Supervisión, DMC, MML, EJA, DO; Administración del proyecto, DMC, EJA, DO; Adquisición de fondos, DO, EZG, EAB.

Declaración de la Junta de Revisión Institucional: El estudio se llevó a cabo de acuerdo con las directrices de la Declaración de Helsinki, y fue aprobado por la Junta de Revisión Institucional (o Comité de Ética) de la Universidad de Colorado (COMIRB, protocolo #21-2551) Universidad del Valle de Guatemala (UVG), y los comités de ética de los CDC; fue financiado por los CDC (CDCGH002243).

Declaración de consentimiento informado: Se obtuvo el consentimiento informado de todos los sujetos involucrados en el estudio.

Declaración de disponibilidad de datos: Los datos presentados en este estudio están disponibles previa solicitud al autor correspondiente. La disponibilidad de algunos de los datos está sujeta a restricciones, debido a la confidencialidad de los participantes, por lo que no se han hecho públicos.

Traducción realizada con la versión gratuita del traductor www.DeepL.com/Translator

Conflictos de interés: DO recibe financiamiento a través de subvenciones de Roche y Sanofi. MML recibe financiamiento a través de una subvención de Roche. EJA recibe financiamiento a través de una subvención de Pfizer, y a través de consultorías de Curevac, Inovio y Moderna.

Referencias

1. Marcela Vélez C, 2021. UNESCO Office Montevideo and Regional Bureau for Science in Latin America and the Caribbean United Nations Educational, Scientific and Cultural Organization (UNESCO).
2. Lazarus JV, Wyka K, White TM, Picchio CA, Rabin K, Ratzan SC, Parsons Leigh J, Hu J, El-Mohandes A, 2022. Revisiting COVID-19 vaccine hesitancy around the world using data from 23 countries in 2021. Nat Commun 13: 3801.
3. Md Khairi, L.N.H.; Fahrni, M.L.; Lazzarino, A.I. The Race for Global Equitable Access to COVID-19 Vaccines. *Vaccines (Basel)* **2022**, *10*, doi:10.3390/vaccines10081306.
4. Larson, H.J.; Gakidou, E.; Murray, C.J.L. The Vaccine-Hesitant Moment. *N Engl J Med* **2022**, *387*, 58-65, doi:10.1056/NEJMra2106441.
5. Das, J.K.; Chee, H.Y.; Lakhani, S.; Khan, M.H.; Islam, M.; Muhammad, S.; Bhutta, Z.A. COVID-19 Vaccines: How Efficient and Equitable Was the Initial Vaccination Process? *Vaccines (Basel)* **2022**, *11*, doi:10.3390/vaccines11010011.
6. Kuehn, M.; LaMori, J.; DeMartino, J.K.; Mesa-Frias, M.; Doran, J.; Korrapati, L.; Bhojwani, R.; Lefebvre, P.; Kirson, N. Assessing barriers to access and equity for COVID-19 vaccination in the US. *BMC Public Health* **2022**, *22*, 2263, doi:10.1186/s12889-022-14636-1.
7. Black, S.; Rappuoli, R. A crisis of public confidence in vaccines. *Sci Transl Med* **2010**, *2*, 61mr61, doi:10.1126/scitranslmed.3001738.
8. MacDonald, N.E.; Hesitancy, S.W.G.o.V. Vaccine hesitancy: Definition, scope and determinants. *Vaccine* **2015**, *33*, 4161-4164, doi:10.1016/j.vaccine.2015.04.036.
9. Domek, G.J.; O'Leary, S.T.; Bull, S.; Bronsert, M.; Contreras-Roldan, I.L.; Bolanos Ventura, G.A.; Kempe, A.; Asturias, E.J. Measuring vaccine hesitancy: Field testing the WHO SAGE Working Group on Vaccine Hesitancy survey tool in Guatemala. *Vaccine* **2018**, *36*, 5273-5281, doi:10.1016/j.vaccine.2018.07.046.
10. Skinner, N.A.; Sanders, K.; Lopez, E.; Sotz Mux, M.S.; Abascal Miguel, L.; Vosburg, K.B.; Johnston, J.; Diamond-Smith, N.; Kraemer Diaz, A. Barriers to COVID-19 vaccine acceptance to improve messages for vaccine uptake in indigenous populations in the central highlands of Guatemala: a participatory qualitative study. *BMJ Open* **2023**, *13*, e067210, doi:10.1136/bmjopen-2022-067210.
11. Olson, D.; Krager, S.; Lamb, M.M.; Rick, A.M.; Asturias, E.J. Knowledge of Norovirus and Attitudes toward a Potential Norovirus Vaccine in Rural Guatemala: A Cross-Sectional Exploratory Survey. *Am J Trop Med Hyg* **2018**, *98*, 1498-1501, doi:10.4269/ajtmh.17-0771.

12. Olson, D.; Rick, A.M.; Krager, S.; Lamb, M.; Asturias, E.J. Vaccine Demand and Willingness-to-pay for Arbovirus Vaccines: A Cross-sectional Survey in Rural Guatemala. *Pediatr Infect Dis J* **2018**, *37*, 1184-1189, doi:10.1097/INF.0000000000002169.
13. Wagner, A.L.; Masters, N.B.; Domek, G.J.; Mathew, J.L.; Sun, X.; Asturias, E.J.; Ren, J.; Huang, Z.; Contreras-Roldan, I.L.; Gebremeskel, B.; et al. Comparisons of Vaccine Hesitancy across Five Low- and Middle-Income Countries. *Vaccines (Basel)* **2019**, *7*, doi:10.3390/vaccines7040155.

14. 2022. Situación de COVID-19 en Guatemala: Ministerio de Salud Pública y Asistencia Social.
15. Taylor L, 2022. Guatemala's COVID vaccine roll-out failed: here's what researchers know. Nature.
16. J V, 2022. Vence último lote de Moderna en Guatemala; 930 mil aún carecen de segunda dosis. La Hora. Guatemala City, Guatemala.
17. AL O, 2022. Se vencen las últimas dosis de Sputnik V, ¿cuántas dosis de otras vacunas nos quedan? Prensa Libre. Guatemala City, Guatemala.
18. Duff, A.; Padilla, A. *Latin America: agricultural perspectives*; September 25, 2015 2015.
19. Representative, O.o.t.U.S.T. Guatemala. Available online: https://ustr.gov/countries-regions/western-hemisphere/guatemala (accessed on
20. Iwamoto, C.; Lesteberg, K.E.; Lamb, M.M.; Calvimontes, D.M.; Guo, K.; Barrett, B.S.; Mickens, K.L.; Duca, L.M.; Monzon, J.; Chard, A.N.; et al. High SARS-CoV-2 Seroprevalence and Rapid Neutralizing Antibody Decline among Agricultural Workers in Rural Guatemala, June 2020-March 2021. *Vaccines (Basel)* **2022**, *10*, doi:10.3390/vaccines10071160.
21. Olson, D.; Calvimontes, D.M.; Lamb, M.M.; Guzman, G.; Barrios, E.; Chacon, A.; Rojop, N.; Arias, K.; Gomez, M.; Bolanos, G.A.; et al. Clinical and Economic Impact of COVID-19 on Agricultural Workers, Guatemala(1). *Emerg Infect Dis* **2022**, *28*, S277-S287, doi:10.3201/eid2813.212303.
22. Asturias, E.J.; Heinrichs, G.; Domek, G.; Brett, J.; Shick, E.; Cunningham, M.; Bull, S.; Celada, M.; Newman, L.S.; Tenney, L.; et al. The Center for Human Development in Guatemala: An Innovative Model for Global Population Health. *Adv Pediatr* **2016**, *63*, 357-387, doi:10.1016/j.yapd.2016.04.001.
23. Olson, D.; Lamb, M.; Lopez, M.R.; Colborn, K.; Paniagua-Avila, A.; Zacarias, A.; Zambrano-Perilla, R.; Rodriguez-Castro, S.R.; Cordon-Rosales, C.; Asturias, E.J. Performance of a Mobile Phone App-Based Participatory Syndromic Surveillance System for Acute Febrile Illness and Acute Gastroenteritis in Rural Guatemala. *J Med Internet Res* **2017**, *19*, e368, doi:10.2196/jmir.8041.
24. Kamidani, S.; Melgar, M.; Robinson, C.; Asturias, E.; Berman, S.; Gaensbauer, J. No Detection of Entamoeba Histolytica by Multiplex Polymerase Chain Reaction in Children With Acute Non-bloody Diarrhea in Guatemala. *Pediatr Infect Dis J* **2018**, *37*, e107-e108, doi:10.1097/INF.0000000000001756.
25. Olson, D.; Calvimontes, D.M.; Lamb, M.M.; Guzman, G.; Barrios, E.; Chacon, A.; Rojop, N.; Arias, K.; Gomez, M.; Bolanos, G.A.; et al. Clinical and Economic Impact of COVID-19 on Plantation Workers: Preliminary Results from the Guatemala Agricultural Workers and Respiratory Illness Impact (AGRI) Study. *medRxiv* **2022**, doi:10.1101/2022.02.07.22270274.

26. 2021. Confianza en la vacuna contra el COVID-19 Guía para la evaluación rápida de la comunidad: Center for Disease Control and Prevention.

27. Patwary, M.M.; Bardhan, M.; Haque, M.Z.; Sultana, R.; Alam, M.A.; Browning, M. COVID-19 Vaccine Acceptance Rate and Its Factors among Healthcare Students: A Systematic Review with Meta-Analysis. *Vaccines (Basel)* **2022**, *10*, doi:10.3390/vaccines10050806.

28. Urrunaga-Pastor D, Bendezu-Quispe G, Herrera-Anazco P, Uyen-Cateriano A, Toro-Huamanchumo CJ, Rodriguez-Morales AJ, Hernandez AV, Benites-Zapata VA, 2021. Cross-sectional analysis of COVID-19 vaccine intention, perceptions and hesitancy across Latin America and the Caribbean. Travel Med Infect Dis 41: 102059.
29. Bates, B.R.; Villegas-Botero, A.; Costales, J.A.; Moncayo, A.L.; Tami, A.; Carvajal, A.; Grijalva, M.J. COVID-19 Vaccine Hesitancy in Three Latin American Countries: Reasons Given for Not Becoming Vaccinated in Colombia, Ecuador, and Venezuela. *Health Commun* **2022**, *37*, 1465-1475, doi:10.1080/10410236.2022.2035943.
30. Kibongani Volet, A.; Scavone, C.; Catalan-Matamoros, D.; Capuano, A. Vaccine Hesitancy Among Religious Groups: Reasons Underlying This Phenomenon and Communication Strategies to Rebuild Trust. *Front Public Health* **2022**, *10*, 824560, doi:10.3389/fpubh.2022.824560.
31. Gehlbach, D.; Vazquez, E.; Ortiz, G.; Li, E.; Sanchez, C.B.; Rodriguez, S.; Pozar, M.; Cheney, A.M. COVID-19 Testing and Vaccine Hesitancy in Latinx Farm-Working Communities in The Eastern Coachella Valley. *Res Sq* **2021**, doi:10.21203/rs.3.rs-587686/v1.

32. Rodriguez-Morales AJ, Franco OH, 2021. Public trust, misinformation and COVID-19 vaccination willingness in Latin America and the Caribbean: today's key challenges. Lancet Reg Health Am 3: 100073.
33. Zarocostas J, 2020. How to fight an infodemic. Lancet 395: 676.
34. Pierri F, Perry BL, DeVerna MR, Yang KC, Flammini A, Menczer F, Bryden J, 2022. Online misinformation is linked to early COVID-19 vaccination hesitancy and refusal. Sci Rep 12: 5966.
